# A Case of Ataxia with Isolated Vitamin E Deficiency Initially Diagnosed as Friedreich's Ataxia

**DOI:** 10.1155/2016/8342653

**Published:** 2016-02-16

**Authors:** Michael Bonello, Partha Ray

**Affiliations:** The Walton Centre NHS Foundation Trust, Lower Lane, Liverpool L9 7LJ, UK

## Abstract

Ataxia with isolated vitamin E deficiency (AVED) is a rare autosomal recessive condition that is caused by a mutation in the alpha tocopherol transfer protein gene. It is almost indistinguishable clinically from Friedreich's ataxia but with appropriate treatment its devastating neurological features can be prevented. Patients can present with a progressive cerebellar ataxia, pyramidal spasticity, and evidence of a neuropathy with absent deep tendon reflexes. It is important to screen for this condition on initial evaluation of a young patient presenting with progressive ataxia and it should be considered in patients with a long standing ataxia without any diagnosis in view of the potential therapeutics and genetic counselling. In this case report we present a patient who was initially diagnosed with Friedreich's ataxia but was later found to have AVED.

## 1. Introduction

Ataxia with isolated vitamin E deficiency (AVED) is a rare autosomal recessive condition that is characterised by progressive cerebellar ataxia, dorsal column signs, and pyramidal deficits on examination. It is caused by a mutation in the alpha tocopherol transfer protein gene on chromosome 8 and was first described by Burck et al. in 1981 in a paediatric patient [1]. Clinically it is almost indistinguishable from Friedreich's ataxia but with appropriate treatment most of its devastating neurological features can be prevented. Below we discuss a patient who had a diagnosis of Friedreich's ataxia but was later found to have AVED.

## 2. Case Presentation

A 28-year-old Iranian male was referred to the regional neurology centre for assessment of his progressive ataxia and dysarthria. He was an asylum seeker that arrived in the UK from Tehran 3 months prior to being seen in clinic. His first symptoms started when he was 15 years old with progressive gait difficulties. He was noted to have wasting of his muscles and paraesthesia of his legs. He developed cerebellar dysarthria 5 years later. He managed to do his first year as a medical student in Iran, but unfortunately due to illness he was unable to complete his studies. He recently became wheelchair bound. Two of his cousins have speech difficulties but no further family history was obtainable. There was no parent consanguinity or gastrointestinal symptoms. Neurological examination confirmed a broad based gait which was clearly ataxic. Bedside examination of the cranial nerves revealed dysarthria, nystagmus, and normal fundoscopy. Upper and lower limb examination confirmed cerebellar ataxia with intention tremor and absent deep tendon reflexes. Plantar responses were extensor on the left and absent on the right. There was absent vibration sense and joint position sense bilaterally. Genetic analysis of the frataxin gene confirmed two alleles in the normal size range and no evidence of an expansion. Further investigation confirmed evidence of vitamin E deficiency. The concentration of alpha tocopherol was measured at <1.0 *μ*mol/L (normal range: 9.5–41.5 *μ*mol/L). The vitamin E/cholesterol ratio was 0.3 *μ*mol/mmol suggesting pure vitamin E deficiency. Other laboratory investigations confirmed a normal full blood count, glucose, liver, kidney function, coeliac antibodies, fasting lipids, thyroid function tests, copper, and ceruloplasmin. Magnetic Resonance Imaging (MRI) of the brain confirmed normal intracranial appearances including no cerebellar atrophy. The diagnosis of AVED was confirmed by mutations in the TTPA gene. There was a homozygous pathogenic frame shift mutation in the TTPA gene* c.706del (p.(His236fs))* which results in loss of activity of the *α*-TTP. The patient started treatment with high dose vitamin E in the form of D-alpha tocopherol supplementation at 800 mg/day. Serum vitamin E concentration improved at 1-year follow-up. His ataxia and dysarthria had stabilised although he was still significantly disabled requiring support in his ADLs.

## 3. Discussion

The role of vitamin E deficiency and neurological disease was first described in 1981 in a 12-year-old with progressive cerebellar ataxia and low serum vitamin E [1]. Further reports followed in the literature until a case series was presented in 1993 of eight patients with Friedreich's ataxia phenotype but low vitamin E [2]. Genetic locus was mapped to chromosome 8q in the same year [3]. In 1995 mutations in the gene encoding hepatic *α*-tocopherol transfer protein (*α*-TTP) were identified in this condition and since then have been termed ataxia with vitamin E deficiency (AVED) [4]. The majority of patients reported in the literature are from Mediterranean countries, particularly North African countries and Japan, although it has been reported in other countries including the United Kingdom [5]. Pathological studies from postmortem examinations of two patients confirmed atrophy of the brainstem, spinal cord, cerebral hemispheres, and cerebellum. There was cell loss in the third cortical layer, giant cells of the striatum, the dentate nucleus, anterior horn cells, neurons of the twelfth and ambiguous nuclei, and the inferior olive. There was Purkinje cell loss in the cerebellum as well as degeneration of the posterior columns and moderate degeneration of the lateral corticospinal tracts [6, 7]. Nerve biopsy shows mild to moderate axonal neuropathy associated with regeneration as opposed to Friedreich's ataxia where the peripheral neuropathy is mainly sensory and severe from the early stages of the disease [8].

*α*-TTP is a liver transfer protein that acts as a carrier molecule for RRR-*α*-tocopherol and binds it preferentially to very low density lipoproteins (VLDL). Thus it acts as an important step in the circulation of RRR-*α*-tocopherol to the nervous system [9]. Vitamin E is an antioxidant and is thought to play a crucial role in neurological function although the specific role is still uncertain. It is thought that oxidative stress in the lack of vitamin E results in damage to the various parts of the nervous system resulting in the clinical signs [10].

The age of onset of clinical signs varies from early childhood to very late adult life [11]. Patients develop a progressive cerebellar ataxia that can lead to severe gait disturbances and disability if not treated early. The type of mutation seems to determine the age of needing a wheelchair, the age of onset, and the progression of the disease [12, 13]. Posterior column involvement is evident in most patients. Romberg's sign is found in most patients and joint position sense is usually impaired. Lateral corticospinal tracts are usually affected late [12]. Pyramidal spasticity is usually evident. Tendon reflexes are normally absent similar to Friedreich's ataxia phenotype. Gaze paralysis and nystagmus often happen. Distal amyotrophy is also noticed in such patients [11]. Other features reported include myoclonus, focal dystonia, deafness, and urinary symptoms [11]. Retinitis pigmentosa is more frequent in Japanese patients and seems to be more prominent with an* H101Q* mutation [14]. Other features include pes cavus and kyphoscoliosis. Cardiac involvement is seen in up to 31% of patients [15].

High dose vitamin E supplementation in patients with AVED results in stabilisation of the neurological features and in some cases can result in improvement [12]. This is an important point which signifies that if treatment is started early, most of the disability could potentially be prevented. This confirms the importance of genetic counselling in patients with AVED.

We advocate that vitamin E levels should form part of the initial screen for a patient with young onset progressive ataxia. In the literature there are a number of cases of patients who have been clinically diagnosed prior to genetic testing being widely available. We do recommend that such patients have their frataxin gene checked and if negative have a vitamin E blood level tested as adequate treatment with high dose vitamin E can halt progression of the condition and can be invaluable in genetic counselling.
